# Evaluation of Two Commercially Available Cannabidiol Formulations for Use in Electronic Cigarettes

**DOI:** 10.3389/fphar.2016.00279

**Published:** 2016-08-29

**Authors:** Michelle R. Peace, Karen E. Butler, Carl E. Wolf, Justin L. Poklis, Alphonse Poklis

**Affiliations:** ^1^Department of Forensic Science, Virginia Commonwealth University, Richmond, VAUSA; ^2^Department of Pathology, Virginia Commonwealth University, Richmond, VAUSA; ^3^Department of Pharmacology and Toxicology, Virginia Commonwealth University, Richmond, VAUSA

**Keywords:** cannabidiol, electronic cigarettes, vaping, e-liquids, DART-MS, HPLC-MS/MS

## Abstract

Since 24 states and the District of Columbia have legalized marijuana in some form, suppliers of legal marijuana have developed *Cannabis sativa* products for use in electronic cigarettes (e-cigarettes). Personal battery powered vaporizers, or e-cigarettes, were developed to deliver a nicotine vapor such that smokers could simulate smoking tobacco without the inherent pathology of inhaled tobacco smoke. The liquid formulations used in these devices are comprised of an active ingredient such as nicotine mixed with vegetable glycerin (VG) and/or propylene glycol (PG) and flavorings. A significant active ingredient of *C. sativa*, cannabidiol (CBD), has been purported to have anti-convulsant, anti-nociceptive, and anti-psychotic properties. These properties have potential medical therapies such as intervention of addictive behaviors, treatments for epilepsy, management of pain for cancer patients, and treatments for schizophrenia. However, CBD extracted from *C. sativa* remains a DEA Schedule I drug since it has not been approved by the FDA for medical purposes. Two commercially available e-cigarette liquid formulations reported to contain 3.3 mg/mL of CBD as the active ingredient were evaluated. These products are not regulated by the FDA in manufacturing or in labeling of the products and were found to contain 6.5 and 7.6 mg/mL of CBD in VG and PG with a variety of flavoring agents. Presently, while labeled as to content, the quality control of manufacturers and the relative safety of these products is uncertain.

## Introduction

Electronic cigarettes (e-cigarettes) were developed as an alternative method for nicotine delivery. Their utility and popularity have transformed them into a general drug-delivery device. They are inexpensive, easy to use, and have some public perception as a healthy alternative. They work by either drawing negative pressure through the mouthpiece or depressing a button to activate a battery that heats a coil, containing a wick saturated with a formulation known as the e-liquid. The e-liquids are made of some ratio of propylene glycol (PG) and vegetable glycerin (VG) and/or a pharmaceutical and/or herbal remedy plus, potentially, a flavoring agent. When the e-cigarette is activated, the e-liquid is vaporized, followed by rapid condensation into an aerosol that the user inhales (Breland et al., 2016; Peace et al., 2016a).

On May 5, 2016, the Food and Drug Administration (FDA) announced the extension of their authority to regulate all tobacco products, including e-cigarettes (U.S. Food and Drug Administration [FDA], 2016). A significant reason to do so was to address the quality assurance of e-cigarette products, from the devices to the nicotine-based e-liquids contained within. The e-liquid formulations have been found to vary significantly from the labeled content around the world (Etter et al., 2013; Goniewicz et al., 2013; Kavvalakis et al., 2015; Pagano et al., 2015; Peace et al., 2016a). Since the legalization of marijuana in some form in 24 states and the U.S. District of Columbia, e-liquids containing cannabinoids have emerged in the market place. As with nicotine e-liquid concentrations, the measured concentration of Δ9-tetrahydrocannabinol (THC) in a commercially available product was found to contain significantly different THC concentration than was labeled (Peace et al., 2016b).

Cannabidiol (CBD) has been purported to have anti-convulsant, anti-nociceptive, and anti-psychotic properties (Brenneisen, 2007; Bhattacharyya et al., 2010). These properties have potential medical therapies such as intervention of addictive behaviors, treatments for epilepsy, management of pain for cancer patients, and treatments for schizophrenia (Johnson et al., 2010; Fischer et al., 2015; Friedman and Devinsky, 2015; Manseau and Goff, 2015). According to the Drug Enforcement Agency (DEA), CBD is a Schedule I substance as defined by the Controlled Substances Act (CSA). Recently, the DEA made it easier for scientists conducting FDA-approved studies to acquire CBD (United States Drug Enforcement Administration [DEA], 2016). Despite the ease of regulation for these research purposes, the CSA still disallows the addition of CBD to products for medicinal benefit since the FDA has not approved it for medical intervention. In 2015 and 2016, the FDA issued warning letters to companies marketing an unapproved drug in their products for therapeutic benefit (United States Food and Drug Administration [FDA], 2015a,b). Some companies selling products containing CBD continue to claim medicinal value for their products. However, some post the FDA disclaimer citing that their products are “not intended for the diagnosis, cure, mitigation, treatment, or prevention of a disease” according to the Federal Food, Drug, and Cosmetic Act and that they do not “distribute any products that are in violation of the U.S. Controlled Substances Act (Cloud 9 Hemp, 2015; Isodiol, 2016).

Aside from volatile organic compounds and other potential degradation products generated by an e-cigarette during aerosolization which may pose health concerns (Flora et al., 2016), condensation aerosols are known to be useful and effective drug delivery systems. If research on CBD demonstrates acceptable therapeutic utility and thereby removed from Schedule I, the inhalation of CBD through an aerosol produced by an e-cigarette may be advantageous over traditional smoking methods and ingestion. THC enriched e-liquid vaporized in an e-cigarette has been demonstrated to be an effective route of administration for cannabinoids (Varlet et al., 2016). Of major import is that these cannabinoid infused e-liquids be subjected to manufacturing standards to ensure safety and quality of product.

Presented is the evaluation of two commercially available e-liquids labeled to contain 3.3 mg/mL CBD in PG and VG with flavorings. The vendor claims that a hemp strain with the highest CBD potency was used in the manufacture of their products (Cloud 9 Hemp, 2015). The products were presumptively evaluated using Direct Analysis in Real Time ion source attached to a time of flight mass spectrometer (DART-MS) for cannabinoids, flavorants, and other possible constituents. Cannabinoids were quantitated by high-performance liquid chromatography–tandem mass spectrometry (HPLC-MS/MS). Alcohols were analyzed by headspace gas chromatography with flame ionization detector (HS-GC-FID). The aerosol produced from the e-liquids with an e-cigarette was analyzed by solid phase microextraction gas chromatograph (SPME-GC/MS).

## Materials

**FIGURE 1:**
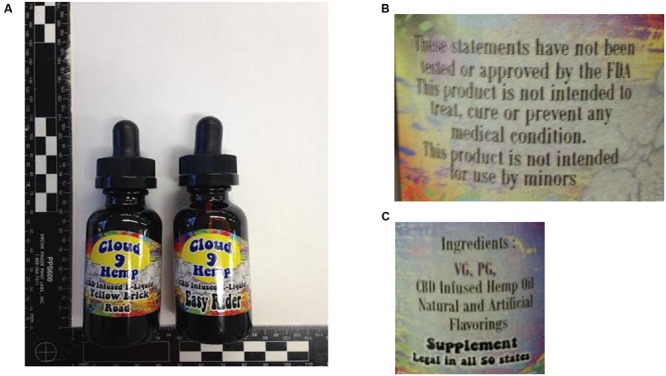
**(A)** Easy Rider and Yellow Brick Road CBD e-liquids with **(B)** the FDA statement regarding the uses of the e-liquid, and **(C)** the listed ingredients of the e-liquids.

Two e-liquids, Cloud 9 Hemp Easy Rider and Yellow Brick Road purported to be infused with CBD, were submitted to the lab for analysis. These products contained no information as to lot number or date of production (**Figure 1**). All tubing, glassware, and fritted gas dispersion tubes were purchased from Colonial Scientific (Richmond, VA, USA). HPLC-grade methanol used for all dilutions, stock and working solutions was purchased from Pharmco-Aaper (Brookfield, CT, USA). Polyethylene glycol (PEG) with an average molecular mass of 600 Da was used for DART-MS calibration and obtained from ULTRA Inc (North Kingstown, RI, USA). United States Pharmacopiea (USP)-grade PG and VG were obtained from Wizard Labs (Altamonte Springs, FL, USA). Nitrogen and helium gases were acquired from Praxair and Airgas (Richmond, VA, USA). Certified ACS Ammonium acetate, formic acid, HPLC-grade methanol and de-ionized (DI) water, optima grade acetone, ethanol, methanol, *n*-propanol, and isopropanol were purchased from Fisher Scientific (Hanover Park, IL, USA). Medical-grade nitrogen and helium were purchased from National Welders Supply Company (Richmond, VA, USA). CBD primary reference standard was purchased from Cerilliant (Round Rock, TX, USA). The e-cigarette was a KangerTech Aerotank clearomizer (v2) attached to an eGo-V v2 variable voltage battery, purchased from 101vape.com (Carlsbad, CA, USA). The single coil was wrapped in non-contact configuration with 34 gauge Nichrome wire to 1.8 Ω and a 2 mm diameter silica string was used as a wick. The flow meter was purchased from Cole Palmer (Vernon Hills, IL, USA). Seven micrometers polydimethylsiloxane (PDMS) SPME fibers were purchased from Supelco (Bellefonte, PA, USA).

## Methods

### Screening of Cannabidiol E-Liquids by DART-MS

A previously published method employing a JEOL JMS T100LC Accu-TOF mass spectrometry controlled by Mass Center software version 1.3.4 m (JEOL Inc., Tokyo, Japan) was used to screen both CBD infused e-liquids for the presence of cannabinoids, flavoring agents, and other possible components (Poklis et al., 2015a,b). Briefly, a capillary tube was dipped five times into an aliquot of each e-liquid prior to introduction into the helium stream of a DART-MS. Each sample was analyzed in positive-ion mode with a helium stream temperature of 300°C. The flow rate was 2.3 L/min with a discharge electrode needle voltage at 150 V and grid electrode at 250 V. The ion guide peak voltage was 400 V, reflectron voltage was 900 V, orifice 2 was set to 5 V, and the ring lens was set to 3 V with orifice 1 operated in function switching mode at 20, 60, or 90 V with a single data file created for all three voltages. The range of masses measured was from 40 to 1,100 Da. Each e-liquid was analyzed five separate times to ensure reproducibility of the results. The data was analyzed by the creation of averaged, background subtracted, centroided mass spectra that was calibrated using the PEG 600. Identification of CBD was made when the exact mass was detected within 5 mDa of its calculated monoisotopic mass (M+H)^+^ and by its fragmentation pattern in function switching mode. PG and VG were confirmed by known standard analysis. All other compounds detected in the e-liquid were identified using a NIST 11.0 library.

### Volatile Analysis of Cannabidiol E-Liquids Headspace GC-FID

A validated analytical method routinely used for the analysis of clinical and forensic samples for ethanol, acetone, isopropanol, and methanol was employed using a Tekmar HT3 headspace sampler attached to a Shimadzu 2014 GC-FID. The chromatographic separation was performed on a RTX-BAC1 (30 m × 0.32 mm id × 1.80 μM column (Restek Corp, Bellefonte, PA, USA). The headspace oven and transfer line temperatures were set to 160°C with a standby flow rate of 200 mL/min. The platen sample temperature was 80°C with the mixer on. The sample equilibrium time was 3.5 min with a sample injection time of 0.5 min. The GC has an oven temperature of 50°C and the injection temperature of 200°C run in split mode with a 1:20 ratio. The column flow rate was 6.85 mL/min with purge flow of 0.5 mL/min, and the detector temperature was 225°C. Calibrators, controls and the CBD e-liquids were diluted with the internal standard, 234 mg/L *n*-propanol in water, at 1:10 ratio prior to analysis. The limit of detection (LOD) for all volatiles was 100 mg/L with a determined linear range of 100–8,000 mg/L for the assay.

### Quantification of Cannabidiol E-Liquids by HPLC-MS/MS

Quantification of CBD was performed using a modification of a previously validated method (Poklis et al., 2010) on an Applied Biosystems 3200 Q Trap with a turbo V source for TurboionSpray (Applied Biosystems, Foster City, CA, USA) tandem mass spectrometer (MS/MS) attached to a Shimadzu SCL HPLC system (Shimadzu Corp., Kyoto, Japan) with a Zorbax eclipse XDBC18 column 4.6 mm × 75 mm, 3.5 μ (Agilent Technologies, Santa Clara, CA, USA) controlled by Analyst 1.4.2 software. The mass spectrometer detector was operated in scan mode with a mass range of 50–700 Da. The injection volume was 10 μL. The mobile phase was 10:90 (v:v) methanol:DI water with a pump flow rate of 0.5 mL/min. The ion spray voltage was 5,000 V with ion source gases 1 and 2 having flow rates of 60 mL/min. The source temperature was set at 650°C and the curtain gas flow rate was 30 mL/min. The following transition ions (*m/z*) were monitored with their corresponding collection energies of 29 eV: CBD: 315 > 193, 315 > 259 and CBD-d_3_: 318 > 196, 318 > 261. The total run time was 8.0 min. Each e-liquid was diluted 1:10,000 and 1:100,000 with methanol. A CBD calibration curve was prepared in duplicate with a range of 10–100 ng/mL. Quality controls specimens of 10, 30, 300, and 750 ng/mL of CBD were prepared in triplicate. A linear regression of the ratio of the peak area counts of cannabinoids and the deuterated internal standards versus concentration was used to construct the calibration curves with a coefficient of determination (*r*^2^) of 0.9995. The transition ions’ relative abundances for the identified cannabinoids were ±20% of target, relative to the calibrators. All quality control specimens were within 15% of their expected target values.

### Analysis of the Aerosol Generated by Cannabidiol E-Liquids

The CBD e-liquids were mixed by hand for 20 s before adding them to a clean KangerTech e-cigarette. The aerosol produced by the e-cigarette was trapped with common glassware. Briefly, two Erlenmeyer flasks were connected in tandem to a vacuum with a flow rate of 2.3 L/min. DI water was added to each trap and a gas dispersion tube bubbled the aerosol into the water. Glass wool was placed in between the two traps to contain the aerosol in the first trap. A 7 μm PDMS SPME fiber was inserted through a stopper in the first trap. The fiber was introduced into the trap while the e-cigarette was activated and the aerosol filled the trap. The e-cigarette was activated for 4 s, aerosolizing 7–10 μL of the e-liquid, and the SPME fiber was held in the trap for 5 min, after which the fiber was retracted. The SPME fiber was inserted into the injection port of an Agilent GC-MS 6890N/5973 Mass Selective Detector (Agilent, Santa Clara, CA, USA) with an HP-5MS column 30 m × 0.25 mm id × 0.25 μm (Agilent, Santa Clara, CA, USA). The injection port was set to 315°C and the run was made in splitless mode with a 15 min thermal desorption time. The initial temperature was set to 120°C, with a ramp of 300°C at 10°C/min, and then a hold time of 12 min, for a total run time of 30 min. Five separate samplings were collected for each e-liquid. The fibers were thermally cleaned between runs following manufacturer specifications to ensure no carryover occurred between samples. A 100 ng/mL CBD reference standard was also analyzed on the GC-MS and the combination of retention time and mass spectrum were used to identify CBD in the aerosolized e-liquids. Flavoring agents were identified using a NIST 11.0 library.

## Results

Cloud 9 Hemp Yellow Brick Road and Easy Rider e-liquids were determined to contain 7.6 and 6.5 mg/L CBD, respectively. Both e-liquids were found to contain PG and VG (65:35 v:v). Yellow Brick Road and Easy Rider e-liquids were also determined to contain 3,600 and 6,600 mg/L ethanol, respectively. Yellow Brick Road was determined to contain the following flavoring agents: peach lactone, y-decalactone, and vanillin, and Easy Rider contained ethyl maltol, ethyl vanillin, vanillin, y-nonalactone, benzaldehyde PG acetal, dimethyl anthranilate, propenylguaethol, 6-methylcoumarin, and heliotropin PG acetal (**Table 1**). The analysis of the aerosol generated using Yellow Brick Road and Easy Rider e-liquids in the KangerTech e-cigarette resulted in the detection of the CBD, PG, VG, and flavoring agents found in the e-liquids (Supplementary Figure S1).

**Table 1:** Compounds detected in the Easy Rider and Yellow Brick Road e-liquids by DART-MS and SPME-GC/MS analysis.

Compound	Formula	Monoisotopic MW (g/mol)	DART-MS [M+H]^+^	SPME-GC/MS RT (minute)
**Easy Rider**				
Ethyl maltol	C7H8O3	140.047	141.061	3:07
Benzaldehyde propylene glycol acetal	C10H12O2	164.084	ND	3:45
Wine ether	C11H22O2	186.162	187.174	3:44
γ-Nonalactone	C9H16O2	156.115	157.123	4:28
Vanillin	C8H8O3	152.047	153.060	4:50
Dimethyl anthranilate	C9H11NO2	165.079	166.091	4.95
Ethyl vanillin	C9H10O3	166.063	ND	5:24
Propenylguaethol	C11H14O2	178.099	179.112	6:05
6-Methylcoumarin	C10H8O2	160.052	161.069*	6:34
Heliotropin propylene glycol acetal	C11H12O4	208.074	209.074	7:14
Benzyl benzeneacetate	C15H14O2	226.099	ND	9:06
Cannabidiol	C21H30O2	314.225	315.232	14:39
**Yellow Brick Road**				
Isomenthol acetate	C12H22O2	198.162	ND	3.82
Vanillin	C8H8O3	152.047	153.062	4:50
Peach lactone	C11H20O2	184.146	185.151	5:29
γ-Decalactone	C10H18O2	170.131	171.141	6:35
Cannabidiol	C21H30O2	314.225	315.232	14:39

MW, molecular weight; ND, not detected by DART-MS.

*Confirmation by GC-MS. Low abundance in DART-MS prohibited identification.

## Discussion

Electronic cigarettes have become a popular means for using pharmaceuticals other than nicotine. This study characterized commercially available e-liquids that were advertised as containing CBD. DART-MS was used to assess the presence of PG, VG, CBD, and a flavor profile for each of the e-liquids. It was also used to evaluate the e-liquids for any other potential constituents. Volatile analysis was used to detect and quantitate the presence of ethanol within the samples. The CBD detected using the DART-MS method was then quantitated using HPLC-MS/MS, and SPME-GC/MS was used to identify compounds aerosolized by the e-cigarette. Only a single cannabinoid, CBD, was detected by the DART-MS, HPLC-MS/MS, and SPME-GC/MS methods.

SPME has historically been used in order to characterize the smoke of traditional cigarettes (Watson et al., 2004). One such study, performed by Bao et al. (2010), used HS-SPME to determine the concentration of free-base nicotine in cigarette smoke. Another study performed by Ye (2008) used SPME-GC/MS in order to analyze any volatile organic compounds found from cigarette smoke from 10 different types of traditional cigarettes. Wu et al. (2002) were also capable of detecting the abundant alkaloids from cigarette smoke from 14 different countries. The present study sought to use a similar concept in order to analyze condensation aerosol of e-liquids containing CBD in order to characterize the aerosol produced by the e-liquids. The e-cigarette aerosolized the CBD as it was successfully extracted from the aerosol using SPME, then thermally desorbed by GC/MS. The final chromatographic profile of the aerosol included PG, VG, CBD, and flavoring agents. No carbonyl compounds were detected in the aerosol produced by the e-liquid using the SPME-GC/MS method presented. These compound have been detected by other investigators in aerosols by other methodologies (Bekki et al., 2014; Kosmider et al., 2014; Flora et al., 2016; Jensen et al., 2015).

While there is no current literature regarding trends among the quality control of CBD containing e-liquids, there have been several involving quantitative studies of nicotine-based e-liquids. Two studies by Goniewicz et al. (2013, 2014) involved the analysis of the concentration of nicotine in cartridges and e-liquids from the UK, Poland, and the United States while Kavvalakis et al. (2015) evaluated products available on the Greek market. Etter et al. (2013), Lisko et al. (2015), and Trehy et al. (2011) analyzed e-liquids and cartridges for flavoring agents, nicotine, and potential nicotine impurities. Pagano et al. (2015) evaluated products purchased in the United States, as did this research group (Peace et al., 2016a). These studies all noted that there was a difference in quantity from the advertised concentration of the active pharmaceutical from the actual concentration, some to be significant. The previous study by Peace et al. (2016b) found that 30% of the e-liquids purchased in the United States had greater than a 20% difference from the labeled concentration of nicotine. This research group also found that a commercially available product, labeled as 69.1% THC and 1.0% CBD, actually contained 42.6% THC (w/v) and 0.5% CBD (w/v). They also found that the THC e-liquid contained four additional cannabinoids and 13 terpenes, indicating that the product was created by extracting *Cannabis sativa* (Peace et al., 2016b). The CBD e-liquids evaluated in this study both contained over twice the advertised concentrations of CBD and also ethanol. Since the e-liquids in this study did not contain any terpenes nor were there any other cannabinoids detected, it could be deduced that the CBD was not produced by an extraction of marijuana.

The detection of the unlabeled ethanol in these products may have been used as a natural flavorant or as a solvent, however, the reason for the ethanol as an ingredient cannot be fully ascertained. The flavorants detected by DART-MS and/or SPME-GC/MS were only included on the product labels as “Natural and Artificial Flavorings.” Lack of regulation provides opportunities for products to be developed without the oversight for the quantity, quality, and safety of products. While they may not necessarily be harmful to the user, it could be important for a consumer, who is trying to monitor their CBD use for medical intervention, to know.

## Conclusion

The e-liquids analyzed in this study both screened positive for CBD by DART-MS and were confirmed to have CBD present by HPLC-MS/MS. SPME was effective in extracting CBD and the other components produced by e-cigarette to confirm aerosolization. The contents of the e-liquids were labeled to contain 3.3 mg/mL of CBD. They were found to contain 6.5 and 7.6 mg/mL of CBD. The analysis of these two different products illustrates the potential quality control issues that can occur in an unregulated industry. Even with uncertain safety of e-cigarettes and e-liquids, the e-cigarette used was shown to generate an aerosol containing CBD using the commercially available e-liquids. Vaping CBD would eliminate harmful combustion products of smoking, while still employing the advantages of inhalation over ingestion. As research continues to understand the effects of CBD and its potential therapeutic properties the e-cigarettes many prove a useful CBD drug delivery system.

## Author Contributions

MP oversees all operations, coordinates experiments and collaborators, and evaluates data to decide quality, experiments, and next directions. KB conducted all experiments on the DART-MS and SPME-GC-MS, significant manuscript preparation. CW conducted the ethanol concentration experiments by GC-MS. JP conducted the CBD experiments by HPLC-MS/MS, significant manuscript preparation. AP coordinated all experiments for ethanol and CBD quantitation, evaluate data, manuscript preparation.

## Conflict of Interest Statement

The authors declare that the research was conducted in the absence of any commercial or financial relationships that could be construed as a potential conflict of interest. The reviewer AD and handling Editor declared their shared affiliation, and the handling Editor states that the process nevertheless met the standards of a fair and objective review.

## Supplementary Material

TABLE S1 **The Total Ion Chromatographs of SPME-GC/MS analysis from the condensation aerosol of the Easy Rider and Yellow Brick Road e-liquids**.
