# Supplementary figures and images for: Evaluation of Two Commercially Available Cannabidiol Formulations for Use in Electronic Cigarettes
Source: Front Pharmacol. 2016 Aug 29;7:279. doi: 10.3389/fphar.2016.00279 (PMC5002419; doi:10.3389/fphar.2016.00279)

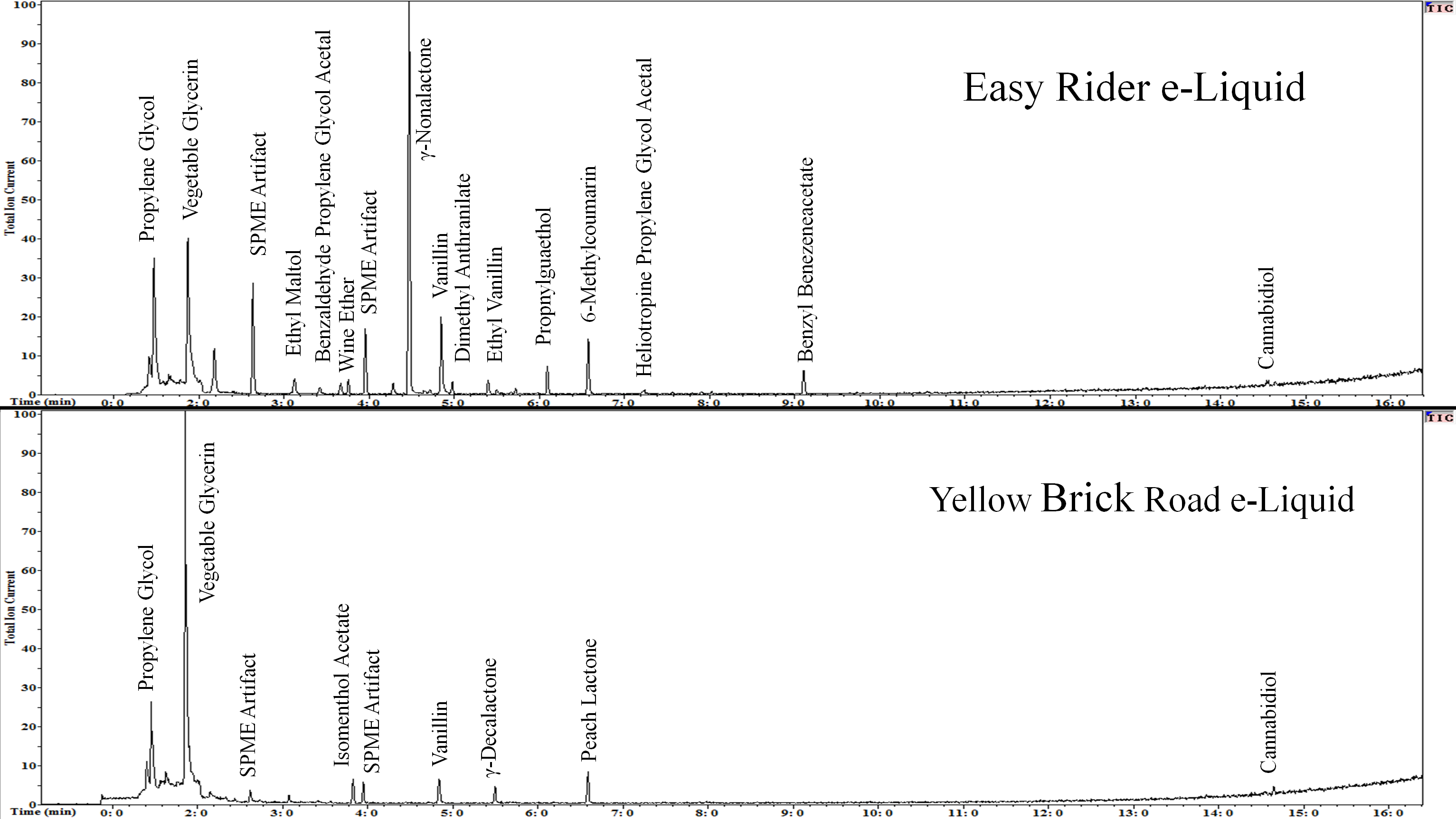

Supplement: TABLE S1 — The Total Ion Chromatographs of SPME-GC/MS analysis from the condensation aerosol of the Easy Rider and Yellow Brick Road e-liquids. [file Image_1.TIF]
